# Surgical Management of Symptomatic Progressive Monostotic Fibrous Dysplasia of the Rib: A Case Report

**DOI:** 10.7759/cureus.101797

**Published:** 2026-01-18

**Authors:** Anita Paiva, Rafael Martins, João Serra, Carlos Pinto, Pedro Fernandes

**Affiliations:** 1 Thoracic Surgery, Unidade Local de Saúde de São João, Porto, PRT; 2 Cardiothoracic Surgery, Unidade Local de Saúde de São João, Porto, PRT

**Keywords:** benign chest wall tumour, monostotic fibrous dysplasia, rib dysplasia, rib resection, thoracic surgery

## Abstract

Fibrous dysplasia is a benign skeletal disorder characterized by replacement of normal bone with fibrous tissue and immature bone, leading to bone fragility, deformity, and pain. The most common presentation is the monostotic form, which is frequently asymptomatic, whereas the polyostotic form is typically more extensive and symptomatic. Management is usually conservative, consisting of clinical and imaging surveillance and pain control, although selected symptomatic cases may benefit from surgical intervention. We report the case of a 33-year-old male who previously underwent partial resection of the left sixth rib due to an expansile bone lesion. Histopathological examination confirmed fibrous dysplasia, and the patient was discharged after one year of follow-up. Fifteen years later, he was referred to thoracic surgery due to persistent left-sided chest pain. Computed tomography demonstrated progression of fibrous dysplasia in the remaining segments of the left sixth rib. Given persistent symptoms, complete surgical excision of the affected rib was performed, resulting in full resolution of symptoms. This case highlights the potential for late disease progression in fibrous dysplasia, even after partial surgical resection, and underscores the importance of long-term follow-up. It also demonstrates that surgical excision can be an effective treatment option in selected symptomatic patients, including those with costal involvement.

## Introduction

Fibrous dysplasia of the bone is an uncommon, benign, non-hereditary skeletal disorder characterized by the replacement of normal bone with fibrous tissue and immature woven bone, resulting in bone fragility, deformity, and occasionally pain or pathological fractures. Clinical presentation is variable, ranging from asymptomatic lesions discovered incidentally to more extensive and symptomatic forms [1]. Fibrous dysplasia can present in two main clinical forms: monostotic and polyostotic, involving one or multiple bones, respectively. The monostotic form is the most common, accounting for approximately 70% of cases. Although any bone can be affected, there is a predilection for long bones, ribs, and craniofacial bones [2,3]. Although the ribs are a recognized site of involvement in fibrous dysplasia, clinically significant costal involvement remains uncommon, and late symptomatic progression after partial resection is rarely reported.

We report the case of a young male with a past medical history of partial resection of the left sixth rib due to fibrous dysplasia, who presented 15 years later with persistent left-sided thoracic pain and radiological findings suggestive of progressive fibrous dysplasia. Due to symptom persistence, complete surgical resection of the remaining rib segment was performed, resulting in full resolution of symptoms.

This case highlights the clinical course and surgical management of symptomatic progressive monostotic fibrous dysplasia of the rib, emphasizing the importance of long-term follow-up after partial resection.

## Case presentation

A 33-year-old male patient with a past medical history of HIV infection, with an undetectable viral load under antiretroviral therapy, was referred to the thoracic surgery outpatient clinic due to left-sided thoracic pain. His symptoms had been present for over six months and were exacerbated by physical exertion, coughing, or sneezing.

**Figure 1:**
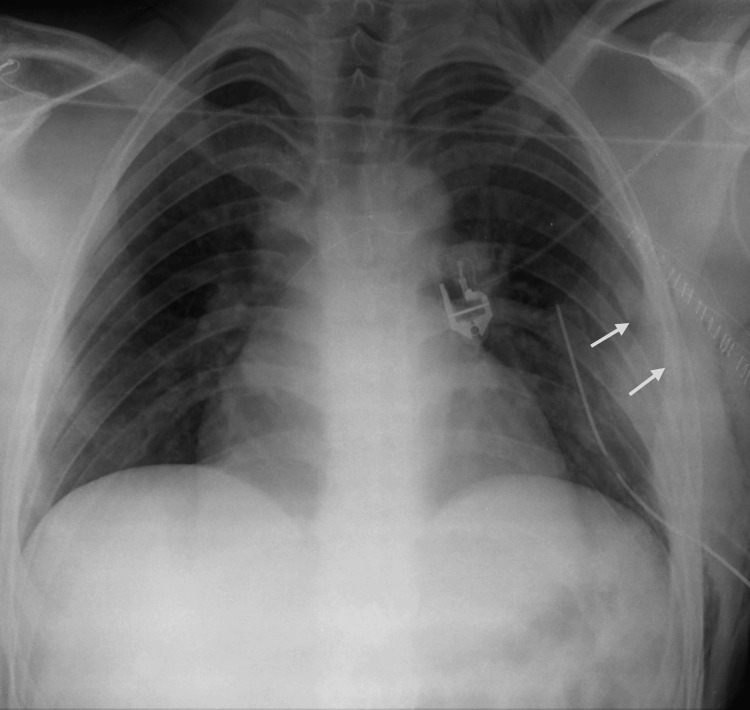
Postoperative chest radiograph after initial partial resection of the left sixth rib Chest radiograph obtained after the initial surgical procedure performed at 18 years of age, demonstrating absence of the middle third of the left sixth rib (arrows), corresponding to the resected segment. A thoracic drain from the initial surgery and skin staples used for closure of the thoracotomy incision are also visible.

At the age of 18, the patient had undergone resection of the middle third of the left sixth rib through a posterolateral thoracotomy for a bone lesion, as documented on chest radiography (Figure 1). According to available medical records at the time of the initial diagnosis, the lesion was confined to the middle segment of the rib. Histopathological examination of the resected specimen revealed a benign fibro-osseous lesion characterized by the absence of cellular atypia and a low mitotic index, with no evidence of malignancy, consistent with a diagnosis of fibrous dysplasia of the rib. After a one-year follow-up period, the patient was discharged from specialized care and referred to his family physician.

**Figure 2:**
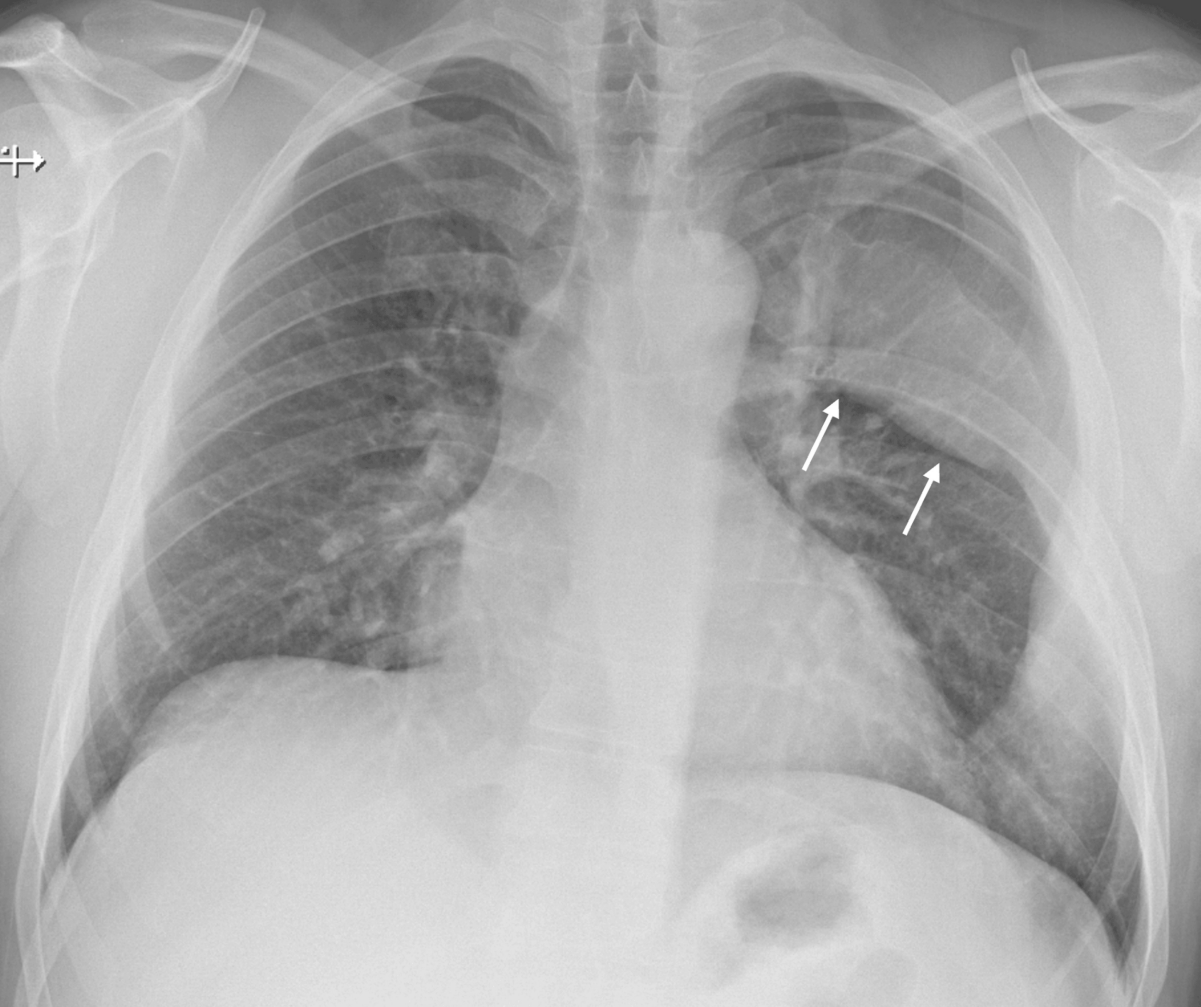
Preoperative chest radiograph Chest radiograph demonstrating an expansile deformity of the left sixth rib with irregular contours (arrows).

**Figure 3:**
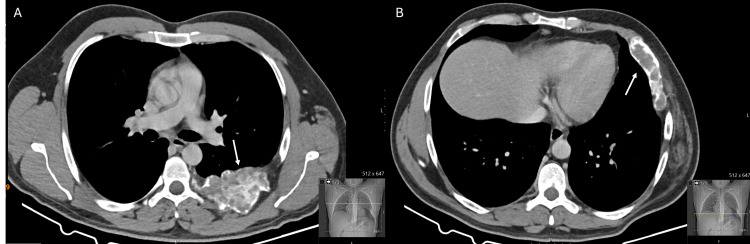
Preoperative chest computed tomography findings Axial computed tomography (CT) images demonstrating fibrous dysplasia involving the posterior (A) and anterior (B) segments of the remaining left sixth rib. Arrows indicate the areas of bone expansion with an abnormal trabecular pattern and cortical thinning, consistent with fibrous dysplasia.

On current presentation, physical examination revealed a hard, non-mobile palpable deformity located below the left nipple, with no additional abnormalities noted. The patient denied any history of malignancy or thoracic trauma. Chest radiography demonstrated an expansile deformity of the left sixth rib with irregular contours (Figure 2). A computed tomography further demonstrated marked dysmorphia of the left sixth rib, with absence of its middle segment, a markedly abnormal trabecular pattern associated with bone expansion, and pronounced cortical thinning. There was no evidence of invasion of adjacent structures (Figure 3).

**Figure 4:**
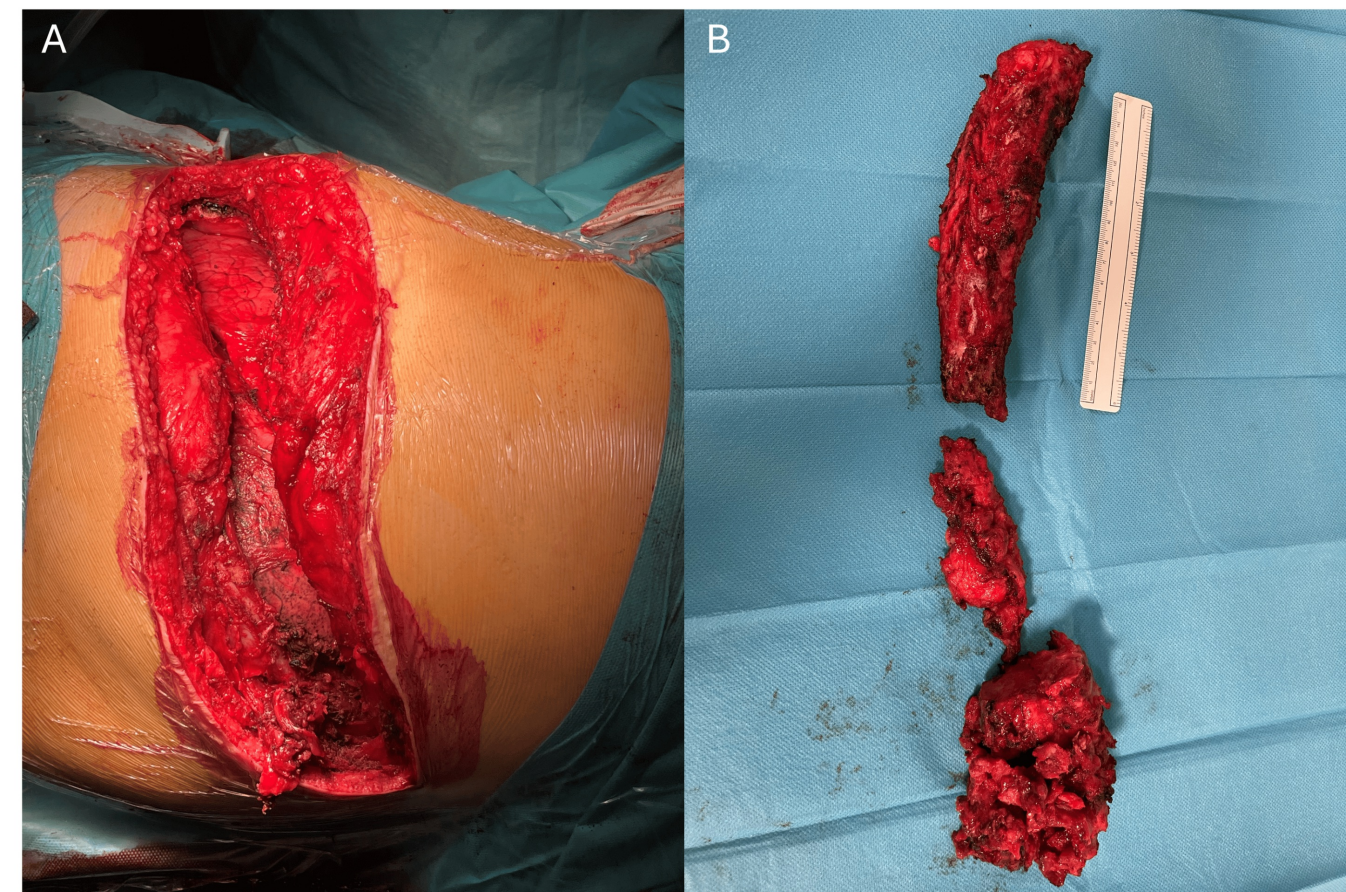
Intraoperative findings during resection of the left sixth rib Intraoperative view following resection of the remaining left sixth rib. (A) Posterolateral thoracotomy after rib excision, with visualization of the left lung and the costal defect corresponding to the resected rib. (B) Resected rib fragments showing dysplastic bone, displayed alongside a 15-cm ruler for size comparison.

Given the patient’s previous history of partial rib resection with histopathological confirmation of fibrous dysplasia involving the same rib, along with compatible radiological findings, progression of residual disease was suspected and surgical management was proposed due to persistent symptoms. An epidural catheter was placed preoperatively for analgesia. A left posterolateral thoracotomy was performed through the sixth intercostal space, using the previous surgical scar as a reference and excising the fibrous tissue from the prior incision. The posterior third of the rib was approached first and dissected up to its insertion at the transverse process, after which the rib head was detached from the transverse process. This stage of the procedure was carried out with the assistance of an orthopaedic surgeon. Subsequently, the anterior third of the rib was approached. The thoracotomy was extended anteriorly to the parasternal region to improve surgical exposure, allowing complete resection of the anterior portion of the rib (Figure 4). At the end of the surgery, one chest tube was placed in the left pleural cavity, as the pleura had been opened during rib dissection.

**Figure 5:**
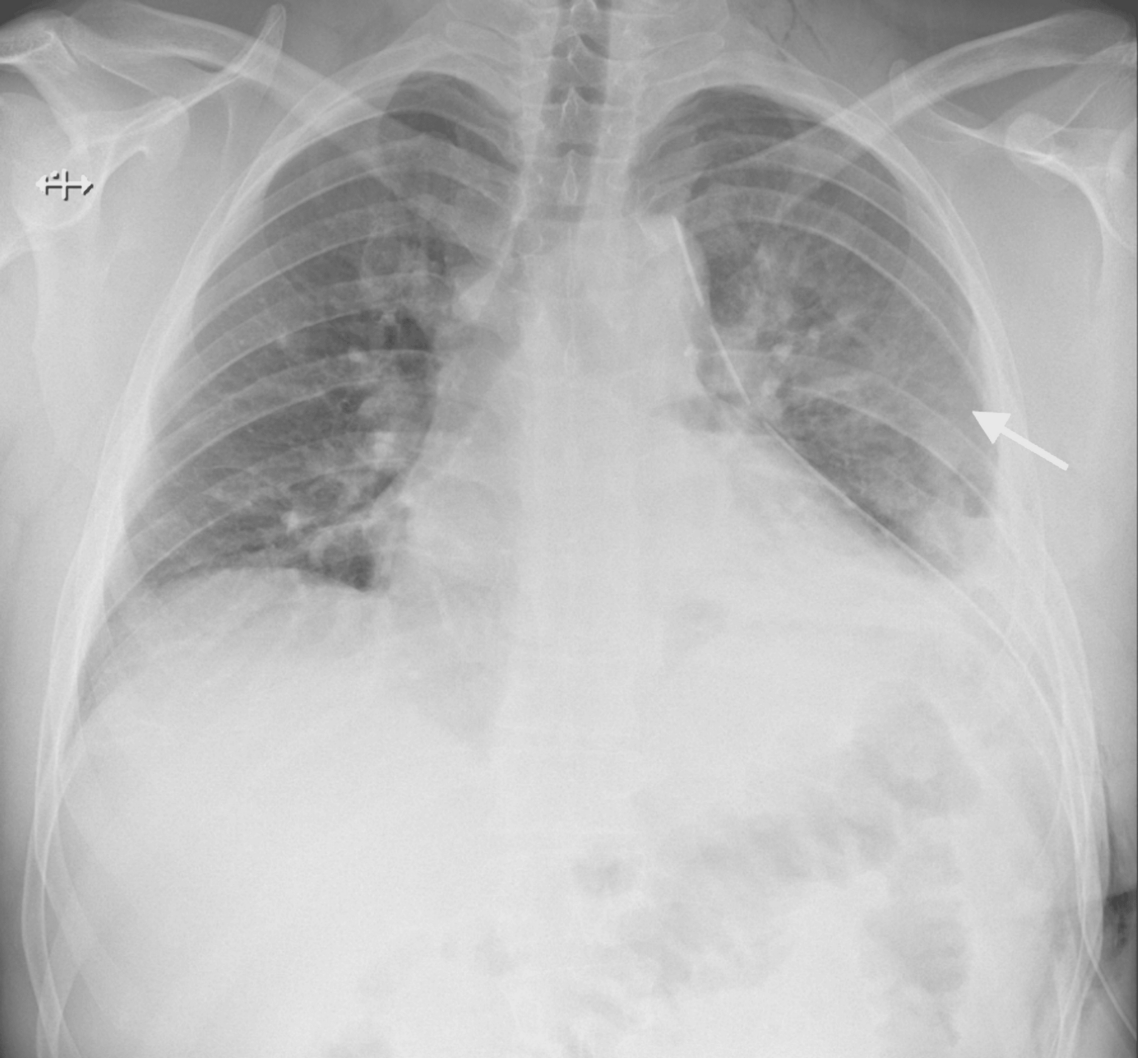
Postoperative chest radiograph after complete resection of the left sixth rib Postoperative chest radiograph demonstrating complete resection of the left sixth rib (arrow) and full expansion of the left lung. A thoracic drain placed during surgery is also visible.

Postoperative chest radiography demonstrated complete expansion of the left lung, absence of pleural effusion, and complete resection of the left sixth rib (Figure 5). The immediate postoperative course was unremarkable, and the patient was discharged home on postoperative day five.

One week after discharge, during outpatient follow-up, the patient developed purulent drainage from the middle third of the surgical wound, associated with fever. Chest radiography showed no evidence of intrathoracic complications. A surgical site infection was clinically suspected, and empirical broad-spectrum antibiotic therapy was initiated and maintained for one week. Following resolution of the infection, negative pressure wound therapy was applied, with no further complications observed. Histopathological examination of the resected specimen confirmed fibrous dysplasia, with no evidence of malignant transformation. At three months postoperatively, the wound had healed completely with a satisfactory aesthetic result, and the patient remained asymptomatic.

## Discussion

Fibrous dysplasia of bone is a benign skeletal disorder caused by activating mutations of the GNAS gene, resulting in abnormal osteoblast differentiation and formation of dysplastic fibro-osseous tissue, with replacement of normal bone by fibrous tissue and immature woven bone. Although any bone can be affected and the ribs represent a recognized site of involvement, rib localization remains relatively uncommon. In monostotic forms, approximately 6-20% of cases involve the ribs. Despite accounting for a significant proportion of benign chest wall tumours, costal fibrous dysplasia remains an infrequent entity in clinical practice [4]. Nevertheless, a small risk of malignant transformation has been described in the literature, most commonly into osteosarcoma, occurring in approximately 0.4-1% of patients and reported more frequently in the polyostotic form [1].

Clinical presentation is variable and depends on the disease form (monostotic or polyostotic) and the bone(s) involved. Most monostotic lesions are asymptomatic and are often detected incidentally on radiographic examinations performed for other indications. In contrast, the polyostotic form involves multiple bones, more commonly presents with pain, deformity, limp, or pathological fractures, and may be associated with cutaneous pigmentation and endocrine dysfunction, as observed in McCune-Albright syndrome [2,5].

Radiographically, the affected bone typically demonstrates bone expansion with a radiolucent, ground-glass appearance, cortical thinning, and absence of a visible trabecular pattern or periosteal reaction [6]. Several lesions may present radiographic features similar to fibrous dysplasia, including ossifying fibromas, osteofibrous dysplasia, giant cell tumours, Langerhans cell histiocytosis, and aneurysmal bone cysts; however, the most important differential diagnosis is low-grade osteosarcoma. In cases in which the diagnosis remains uncertain based on clinical or imaging features, or when there is concern for possible malignancy, a biopsy may be performed for histologic evaluation and genetic analysis, including testing for causative GNAS mutations [7,8].

There is no curative treatment for fibrous dysplasia. Management depends on the patient’s age, lesion location and extent, and disease activity, and most patients are managed conservatively. Medical treatment is primarily directed toward pain control. Bisphosphonates may have a role in alleviating bone pain due to their antiresorptive activity; however, there is no evidence that they reduce disease activity or lesion progression. Surgical management is reserved for patients with symptoms or complications related to fibrous dysplasia, such as pain refractory to medical treatment, pathological fractures or high fracture risk, lesions involving adjacent structures, or progressive deformity. In certain locations, such as the ribs, surgical resection represents a therapeutic option. Long-term follow-up is essential to evaluate disease progression or recurrence and to monitor potential complications. Follow-up is based on clinical and imaging assessment, as there are no well-defined guidelines regarding surveillance intervals [1,9].

This case illustrates disease progression in the remaining dysplastic bone following partial resection, with the initial surgical approach appropriately tailored to the extent of disease at that time. It highlights the importance of long-term follow-up and underscores the role, applicability, and favourable outcomes of surgical management in symptomatic cases of fibrous dysplasia, particularly in less commonly reported locations such as the ribs.

## Conclusions

Fibrous dysplasia is a benign condition with a variable clinical course. Management is conservative in most cases; however, surgical intervention has a role in selected symptomatic patients, with favorable outcomes following excision in certain locations, such as the ribs. This case highlights that partial resection does not necessarily prevent later disease progression, underscoring the importance of long-term follow-up, as residual dysplastic bone may continue to evolve over time.
